# Occurrence and distribution of *Giardia* species in wild rodents in Germany

**DOI:** 10.1186/s13071-018-2802-z

**Published:** 2018-03-27

**Authors:** Yosra A. Helmy, Nastasja G. Spierling, Sabrina Schmidt, Ulrike M. Rosenfeld, Daniela Reil, Christian Imholt, Jens Jacob, Rainer G. Ulrich, Toni Aebischer, Christian Klotz

**Affiliations:** 1 Unit 16 Mycotic and Parasitic Agents and Mycobacteria, Department of Infectious Diseases, Robert Koch-Institute, 13353 Berlin, Germany; 2 Institute of Novel and Emerging Infectious Diseases, Friedrich-Loeffler-Institut, Federal Research Institute for Animal Health, 17493 Greifswald - Insel Riems, Germany; 3 Institute for Plant Protection in Horticulture and Forests, Vertebrate Research, Julius Kühn-Institute, Federal Research Centre for Cultivated Plants, 48161 Münster, Germany; 4 Department Panel Veterinary Public Health, Department of Veterinary Medicine, Freie Universität Berlin, 14163 Berlin, Germany; 5 Department of Animal Hygiene, Zoonoses and Animal Ethology, Faculty of Veterinary Medicine, Suez Canal University, Ismailia, 41511 Egypt; 6 German Society of Tissue Transplantation, Kruppstraße 98, 45145 Essen, Germany

**Keywords:** *Giardia* spp., Protozoan infection, Sequence typing, Wild rodents, Reservoir

## Abstract

**Background:**

Giardiasis is an important gastrointestinal parasitic disease in humans and other mammals caused by the protozoan *Giardia duodenalis*. This species complex is represented by genetically distinct groups (assemblages A-H) with varying zoonotic potential and host preferences. Wild rodents can harbor potentially zoonotic assemblages A and B, and the rodent-specific assemblage G. Other *Giardia* spp. found in these animals are *Giardia muris* and *Giardia microti*. For the latter, only limited information on genetic typing is available. It has been speculated that wild rodents might represent an important reservoir for parasites causing human giardiasis. The aim of this study was to investigate the occurrence and distribution of *Giardia* spp. and assemblage types in wild rodents from different study sites in Germany.

**Results:**

Screening of 577 wild rodents of the genera *Apodemus*, *Microtus* and *Myodes*, sampled at eleven study sites in Germany, revealed a high overall *Giardia* prevalence. *Giardia* species determination at the *SSU* rDNA gene locus revealed that *Apodemus* mice, depending on species, were predominantly infected with one of two distinct *G. muris* sequence types. *Giardia microti* was the predominant parasite species found in voles of the genera *Microtus* and *Myodes*. Only a few animals were positive for potentially zoonotic *G. duodenalis*. Subtyping at the beta-giardin (*bg*) and glutamine dehydrogenase (*gdh*) genes strongly supported the existence of different phylogenetic subgroups of *G. microti* that are preferentially harbored by distinct host species.

**Conclusions:**

The present study highlights the preference of *G. muris* for *Apodemus*, and *G. microti* for *Microtus* and *Myodes* hosts and argues for a very low prevalence of zoonotic *G. duodenalis* assemblages in wild rodents in Germany. It also provides evidence that *G. muris* and *G. microti* subdivide into several phylogenetically distinguishable subgroups, each of which appears to be preferentially harbored by species of a particular rodent host genus. Finally, the study expands the database of sequences relevant for sequence typing of *G. muris* and *G. microti* isolates which will greatly help future analyses of these parasites’ population structure.

**Electronic supplementary material:**

The online version of this article (10.1186/s13071-018-2802-z) contains supplementary material, which is available to authorized users.

## Background

Giardiasis caused by the flagellated protozoan *Giardia duodenalis* (syn. *G. lamblia* and *G. intestinalis*) is one of the most frequent gastrointestinal parasitic diseases in humans worldwide [1, 2]. *Giardia duodenalis* is considered as a species complex of genetically different but morphologically identical organisms with varying zoonotic potential and host preferences. Humans and a wide range of mammal species including livestock, pets and wildlife are susceptible to these parasites [3]. This species complex consists of at least eight genetically distinct groups (referred to as assemblages A to H) based on sequence analyses of a few genomic markers such as glutamate dehydrogenase (*gdh*), beta-giardin (*bg*) and small-subunit rRNA (*SSU* rDNA) genes [4–9]. It has been suggested to consider part of these assemblages as distinct *Giardia* species due to their genetic distance and differing host preferences [3]. *Giardia duodenalis* assemblages A and B show the broadest host range, which includes humans and, therefore, they are considered potentially zoonotic [3, 8, 10]. Assemblages C-H are mainly found in certain mammal taxa: C and D in canids, E in ruminants, F in felids, G in rodents, and H in marine mammals [3, 11]. The overall impact of zoonotic transmission on the epidemiology of human infections remains unclear [3].

Besides *G. duodenalis*, to date two more *Giardia* species, *Giardia microti* and *Giardia muris*, are known to infect mammals. Both species are found in small rodents with supposedly different host preferences. *Giardia microti* is thought to be a parasite mainly of rodents belonging to the family Cricetidae such as voles and muskrats and *G. muris* mainly of rodents belonging to the family Muridae [3, 12–15]. Thus, wild rodents can be infected with different *Giardia* species, including *G. microti*, *G. muris* and various assemblages of *G. duodenalis* (assemblages A, B and G) [3, 15–18]. To evaluate the epidemiology and the potential zoonotic risk, molecular surveys for *Giardia* spp. in various wild rodents and lagomorphs, including the North American beaver (*Castor canadensis*), muskrat (*Ondatra zibethicus*), brown rat (*Rattus norvegicus*), house mouse (*Mus musculus*) and prairie vole (*Microtus ochrogaster*), have been attempted to determine the *Giardia* species or assemblage types. However, systematic molecular studies are still comparatively rare, in particular if one considers the broad range of existing rodent host species [8, 9, 13, 15, 17–20]. In fact, earlier studies suggested a link of waterborne human giardiasis outbreaks to a source in wildlife, in particular beavers, that led to the classification of *Giardia* as a zoonotic agent [21, 22]. However, the distribution of different *Giardia* species in rodents of various genera and their geographical distribution based on molecular studies is not sufficiently clarified [21].

Rodents can carry a multitude of pathogens, including important zoonotic viruses, bacteria and parasites [23–25] and as infections with *Giardia* spp. are common in wild rodents it has been discussed whether they could play an important role as a reservoir for these potentially zoonotic parasites as well [17, 19, 24, 26].

The goal of this study was to investigate the occurrence and distribution of *Giardia* spp., including *Giardia* species allocation, in wild rodents from different study sites in Germany.

## Results

### Frequent occurrence of *Giardia* spp. in wild rodents in Germany

Fecal samples of 577 small mammals, collected between 2011 and 2012, were included in the analysis. Samples were collected at eleven sampling sites across four federal states of Germany (Additional file 1: Figure S1, Table 1). Sampling sites fell into five distinct geographical regions (Additional file 1: Figure S1). Sampled animals belonged to six species of three rodent genera: *Apodemus* [*n* = 93, 16%; including *Apodemus agrarius* (striped field mouse), *Apodemus flavicollis* (yellow-necked mouse) and *Apodemus sylvaticus* (wood mouse)]; *Microtus* [*n* = 181, 31%; including *Microtus agrestis* (field vole) and *Microtus arvalis* (common vole)]; and *Myodes* [*n* = 303, 52%; represented by *Myodes glareolus* (bank vole)]; see Table 1 for further details and local distribution.

**Table 1:** *Giardia* spp. in wild rodents as determined by various detection methods for samples from different study sites

Species	Analysis	Rodent samples by site (sample size/*Giardia* detection)											Total	*Giardia* detection	Prevalence (%)^c^ (95% CI)^d^
		Billerbeck^a^	Gotha^b^	Krahnberg^b^	Schaderode^a^	Jeeser	Saal	Kammerforst^a^	Leinawald^b^	Pahnaer Holz^b^	Phoenix Ost	Weissach^a,b^			
*Apodemus agrarius*	IFA		4/2	1/1		17/3		7/1	2/0	2/0	2/1		35	8	22.9 (10.4–40.1)
	qPCR		4/2	1/0		17/7		7/5	2/1	2/2	2/1		35	18	51.4 (34.0–68.6)
	*SSU* PCR		4/2	1/1		17/14		7/3	2/0	2/2	2/2		35	24	68.6 (50.7–83.1)
*Apodemus flavicollis*	IFA	6/1	3/0			7/5	1/1	5/1				16/1	38	9	23.7 (11.4–40.2)
	qPCR	6/0	3/1			7/5	1/0	5/5				16/1	38	12	31.6 (17.5–48.6)
	*SSU* PCR	6/2	3/1			7/5	1/1	5/1				16/3	38	13	34.2 (19.6–51.3)
*Apodemus sylvaticus*	IFA	4/0		2/2	2/2							1/1	9	5	55.6 (21.2–86.3)
	qPCR	4/0		2/0	2/0							1/0	9	0	0 (0–33.6)
	*SSU* PCR	4/0		2/1	2/0							1/1	9	2	22.2 (2.8–60.0)
*Microtus agrestis*	IFA		11/10	13/10		6/5		11/11	10/9	8/7	6/5		65	57	87.7 (77.2–94.5)
	qPCR		10/9	8/8		6/6		11/11	10/10	8/8	6/6		59	58	98.3 (90.9–100)
	*SSU* PCR		11/10	8/7		6/4		11/9	10/10	8/7	6/5		60	52	86.7 (75.4–94.1)
*Microtus arvalis*	IFA	1/1	63/55	2/2	9/7	18/16		1/1	1/1			13/13	108	96	88.9 (81.4–94.1)
	qPCR	1/1	63/58	2/2	8/7	18/18		1/1	1/1			13/9	107	97	90.7 (83.5–95.4)
	*SSU* PCR	1/1	63/52	2/2	8/7	18/13		1/1	1/1			13/9	107	81	75.7 (66.5–82.5)
*Myodes glareolus*	IFA	74/47	61/49	2/2		73/60	2/2	7/6	10/9		1/1	73/63	303	239	78.9 (73.8–83.3)
	qPCR	74/64	61/58	2/2		73/68	2/2	7/7	10/9		1/1	73/63	302	274	90.7 (86.9–93.8)
	*SSU* PCR	74/40	61/47	2/2		72/56	2/2	7/5	10/9		1/1	72/35	301	191	63.5 (57.7–68.9)

^a^Discrimination of *A. flavicollis* and *A. sylvaticus* by molecular typing was not successful in Billerbeck for four animals (all *Giardia-*negative by IFA), in Schaderode for three animals (all *Giardia* positive by IFA), in Kammerforst for three animals (all *Giardia-*negative by IFA) and in Weissach for one animal (*Giardia* negative by IFA). These animals were excluded from the analysis in the table

^b^Discrimination of *Microtus* spp. by molecular typing was not successful in Gotha for one animal (*Giardia* positive by IFA), in Krahnberg for three animals (two *Giardia* positive by IFA), in Leinawald for two animals (all *Giardia* positive by IFA), in Pahnaer Holz for one animal (*Giardia* negative by IFA) and in Weissach for one animal (*Giardia* positive by IFA). These animals were excluded from the analysis in the table

^c^Here the term prevalence is used to describe the proportion of *Giardia* spp. infections in the analyzed animal samples and is not meant to be understood as the ‘real’ prevalence of entire populations

^d^Test for any difference of *Giardia* prevalence from rodent category was done using Fisher-Freeman-Halton test, IFA (*P* ≤ 0.0001), qPCR (*P* ≤ 0.0001), *SSU* PCR (*P* ≤ 0.0001). Comparison between groups was done by using Fisher’s exact test followed by multiple testing correction (Bonferroni-Holm procedure) and *P-*values are presented in Additional file 2: Table S1

All samples were initially tested by immunofluorescence assay (IFA) and cyst-like particles reacting with cyst-wall specific antibodies were found in 423 of 577 samples (73%, 95% confidence interval, 95% CI: 70–77%). The prevalence as revealed by the IFA analysis was significantly different between the genera *Microtus* (159 of 181 samples, 88%, 95% CI: 82–92%), *Myodes* (239 of 303 samples, 79%, 95% CI: 74–83%) and *Apodemus* (25 of 93 samples, 27%, 95% CI; 18–37%) (adjusted Fisher’s exact test *P*-values: *Microtus vs Myodes P* = 0.014; *Microtus vs Apodemus P* < 0.0001; *Myodes vs Apodemus P* < 0.0001). Differences among species of the same genus or among the same species from different study sites were not observed or could not be tested appropriately due to low sample size (Table 1, Additional file 2: Table S1).

Subsequently DNA was extracted and analyzed by previously described real time-PCR (qPCR) [27] and semi-nested PCR assays (*SSU*-PCR) [4, 28] targeting the *SSU* rDNA gene locus. The respective number of samples yielding specific PCR products was 471 of 569 (83%, 95% CI: 79–86%) for the qPCR and 371 of 569 (65%, 95% CI: 61–69%) for the *SSU*-PCR assay. Of note, in all three rodent genera a higher number of infected animals were detected by qPCR compared to IFA analyses, possibly due to higher sensitivity of the former assay as previously suggested [27, 29] (Table 1). The qPCR and *SSU*-PCR tests overall confirmed the higher *Giardia* prevalence in *Microtus* and *Myodes* compared to *Apodemus* (Table 1 and data not shown).

Since samples of *Apodemus* spp. were less frequently positive than those of *Microtus* and *Myodes*, we estimated parasite abundance to test whether a lower abundance could in part explain this finding. For this purpose we performed a semi-quantitative analysis of the cyst numbers in fecal samples using the IFA data and of parasite DNA abundance in feces using the threshold cycle (Ct) value of the qPCR results. The latter served as a proxy for parasite numbers. DNA samples of *Apodemus* feces showed significantly higher Ct-values when amplifying *Giardia SSU* rDNA compared to samples from *Myodes* (delta Ct ~ 2; *P* < 0.001) and *Microtus* (delta Ct ~ 4; *P* < 0.001) (Table 2). This implies on average 4–16 times less *Giardia* DNA content in *Apodemus* samples compared to those of *Myodes* and *Microtus* (Table 2) and, most likely, reflects the lower abundance of parasite cysts in *Apodemus* animals confirmed by semi-quantitative analysis of IFA data (Table 2). The qPCR Ct-values for *Myodes* and *Microtus* were also significantly different, but such a difference was not noted at the resolution of the semi-quantitative IFA test (Table 2). Thus, *Giardia* burden is likely to differ among rodents of different genera and decreasing when comparing *Microtus* and *Myodes vs Apodemus*.

**Table 2:** Relative *Giardia* abundance in rodent samples. Relative *Giardia* abundance in rodent samples was determined by semi-quantitative IFA and by analysis of ct-values of *Giardia* positive samples in a *Giardia* specific qPCR assay^a^

Genus	Species	Ct values (qPCR)			Semi-quantitative cyst abundance (IFA)^c^ absolute numbers (%) [95% CI]			
		Median (Range)^b^	95% CI	*n*	+	++	+++	*n*
*Apodemus*	*agrarius*	33.9 (23.5–38.1)	32.2–35.4	18	8 (100) [63.1–100]	0 (0) [0–36.9]	0 (0) [0–36.9]	8
	*flavicollis*	32.9 (23.9–39.9)	30.7–35.6	12	6 (86) [42.1–99.6]	1 (14) [0.4–57.9]	0 (0) [0–40.0]	7
	*sylvaticus*	no ct			5 (100) [47.8–100]	0 (0) [0–52.2]	0 (0) [0–52.2]	5
Total^d^		33.4 (23.5–39.9)	32.4–34.6	34	22 (96) [78.1–99.8]	1 (4) [0–21.9]	0 (0) [0–14.8]	23
*Microtus*	*agrestis*	28.1 (22.9–36.3)	27.5–29.1	58	43 (80) [66.5–89.4]	10 (19) [9.3–31.4]	1 (2) [0.1–12.3]	54
	*arvalis*	29.0 (20.9–38.3)	28.4–30.0	97	57 (58) [47.2–67.5]	28 (28) [19.7–38.2]	14 (14) [8.0–22.6]	99
Total^d^		28.5 (20.9–38.3)	28.4–29.5	163	104 (70) [62.7–77.9]	25 (17) [11.3–24.1]	18 (12) [7.4–18.6]	147
*Myodes*	*glareolus*	30.7 (22.8–39.9)	30.4–31.2	274	149 (64) [57.7–70.4]	50 (21) [16.4–27.4]	33 (14) [10.0–19.4]	232

*Abbreviations*: *Ct* threshold cycle, *IFA* immunofluorescence assay, *n* sample size, *qPCR* real-time PCR, *95% CI* 95% confidence interval

^a^One should note that parasite excretion is often not uniform. However, it is assumed that such effects averaged out by analyzing the means of different groups

^b^Significant differences of median-values analyzed by non-parametric Kruskal-Wallis test (H = 53.8, *P* < 0.0001). *Post-hoc* test, Dunn’s test of multiple comparisons using rank sums: Ct-values were different between genera (*Apodemus vs Microtus P* < 0.0001, *Apodemus vs Myodes P* < 0.0001, *Microtus vs Myodes P* < 0.0001; mean ranks for *Apodemus*= 353.9, for *Microtus*=183.5 and for *Myodes*=252.6). There were no significant differences within the species of the same genus

^c^Fecal samples were distributed on slides using inoculation loop (*~* 10 μl) and analyzed by IFA. Cyst amount of the samples were semi-quantified (1–10 cysts (+), 11–50 cysts (++), > 50 cysts (+++). Comparison of (+) *vs* (++/+++) between groups was done using Fisher’s exact test followed by multiple testing correction (Bonferroni-Holm procedure): *Apodemus vs Microtus* (*P* = 0.019); *Apodemus vs Myodes* (*P* = 0.005); *Microtus vs Myodes* (*P* = 0.218)

^d^Animals for which only genus could be determined were included

### *Giardia muris* and *G. microti* predominate in wild rodents while zoonotic *Giardia* spp. were rarely detected

To determine the *Giardia* species in wild rodents the sequences of 371 (of *n* = 571 investigated) PCR products of the semi-nested *SSU* PCR (see above) were determined, analyzed and compared to reference sequences (Table 3). Overall, 358 sequences could be typed and 355 of these sequences could be assigned to *Giardia* spp. and three sequences to *Octomitus intestinalis*, a sister lineage of *Giardia* spp. [30].

**Table 3:** PCR and typing results of the semi-nested PCR at the *SSU* RNA gene locus (*SSU* PCR)

Animals		Typed	*Giardia microti*		*Giardia muris*		*Giardia duodenalis* ^d^		*Octomitus intestinalis*		Non-typeable	Neg. (PCR)
Genus	Species	No. of positive samples (*n*)	*n* (%)	95% CI (%)	*n* (%)	95% CI (%)	*n* (%)	95% CI (%)	*n* (%)	95% CI (%)	*n*	*n*
*Apodemus*	*agrarius*	24 (35)	2		21						1	11
	*flavicollis*	13 (38)	5		8							25
	*sylvaticus*	2 (9)			2							7
Total^e^		39 (93)	7 (17.9)^a, b^	9.3–36.5	31 (79.5)^a, b^	60.7–88.9	1 (2.6)	0.6–13.5			1	53
*Microtus*	*agrestis*	52 (60)	49		2				1			8
	*arvalis*	81 (107)	78						1		2	26
Total^f^		138 (175)	134 (97.1)^a, c^	92.7–99.2	2 (1.4) ^a, c^	0.2–5.1			2 (1.4)	0.2–5.1	2	35
*Myodes*	*glareolus*	181 (301)	173 (95.6)^b, c^	91.5–98.1	3 (1.7)^b, c^	0.3–4.8	4 (2.2)	0.6–5.6	1 (0.6)	0.01–3.0	10	110
Total		358 (571)	314 (87.7)	83.9–90.9	36 (9.8)	6.9–13.3	5 (1.4)	0.5–3.2	3 (0.8)	0.2–2.4	13	200

*Abbreviations*: *n* sample size, *CI* confidence interval, neg negative

^a-c^Test for any difference of proportions (*G. microti*, *G. muris*) from rodent category (*Apodemus*, *Microtus*, *Myodes*) was done using Fisher-Freeman-Halton test, *P*-value < 0.0001. Comparison between groups was done by using Fisher’s exact test followed by multiple testing correction (Bonferroni-Holm procedure): ^a^*Apodemus vs Microtus*, *P-*value (adjusted) < 0.0001. ^b^*Apodemus vs Myodes*, *P-value* (adjusted) < 0.0001. ^c^*Microtus vs Myodes*, *P-*value (adjusted) = 1

^d^*G. duodenalis* assemblage A was found in 3 animals (1 *Apodemus*, 2 *Myodes*), *G. duodenalis* assemblage B was found in 2 animals (both *Myodes*)

^e^Including 6 animals for which *A. flavicollis* and *A. sylvaticus* could not be discriminated

^f^Including 8 animals for which *Microtus* spp. could not be discriminated

The analysis revealed that 97% of *Microtus* and 96% of *Myodes* were infected with *G. microti*. *Apodemus* spp. were predominantly infected with *G. muris* (80%) but a sizeable fraction contained *G. microti* (18%) (see Table 3 for details). There were apparent differences in the *G. muris/G. microti* proportion between *A. agrarius* and *A. flavicollis*, but absolute numbers of samples from these two species were too low to allow meaningful conclusions (Table 3).

Only five samples (of *n* = 358, 1.4%; Table 3) were found positive for *G. duodenalis*, and respective sequences could be assigned to assemblage A (in case of one *Apodemus*, two *Myodes* samples) and B (in two samples from *Myodes*). Attempts to further subtype these samples at different genomic loci (*tpi*, *bg* and *gdh*) were not successful (data not shown).

Of the three *O. intestinalis*-positive samples (two from *Microtus*, one from *Myodes*) two were positive in the IFA analysis and all samples were also positive in the *Giardia*-specific qPCR analysis. Further subtyping at *bg* locus (see below) from the two IFA positive samples revealed *G. microti* as a result indicating possible *G. microti*/*O. intestinalis* co-infections in these animals.

### Sequence analysis of *SSU* rDNA, *bg* and *gdh* genes revealed high variation in *G. microti* and *G. muris*

**Fig. 1:**
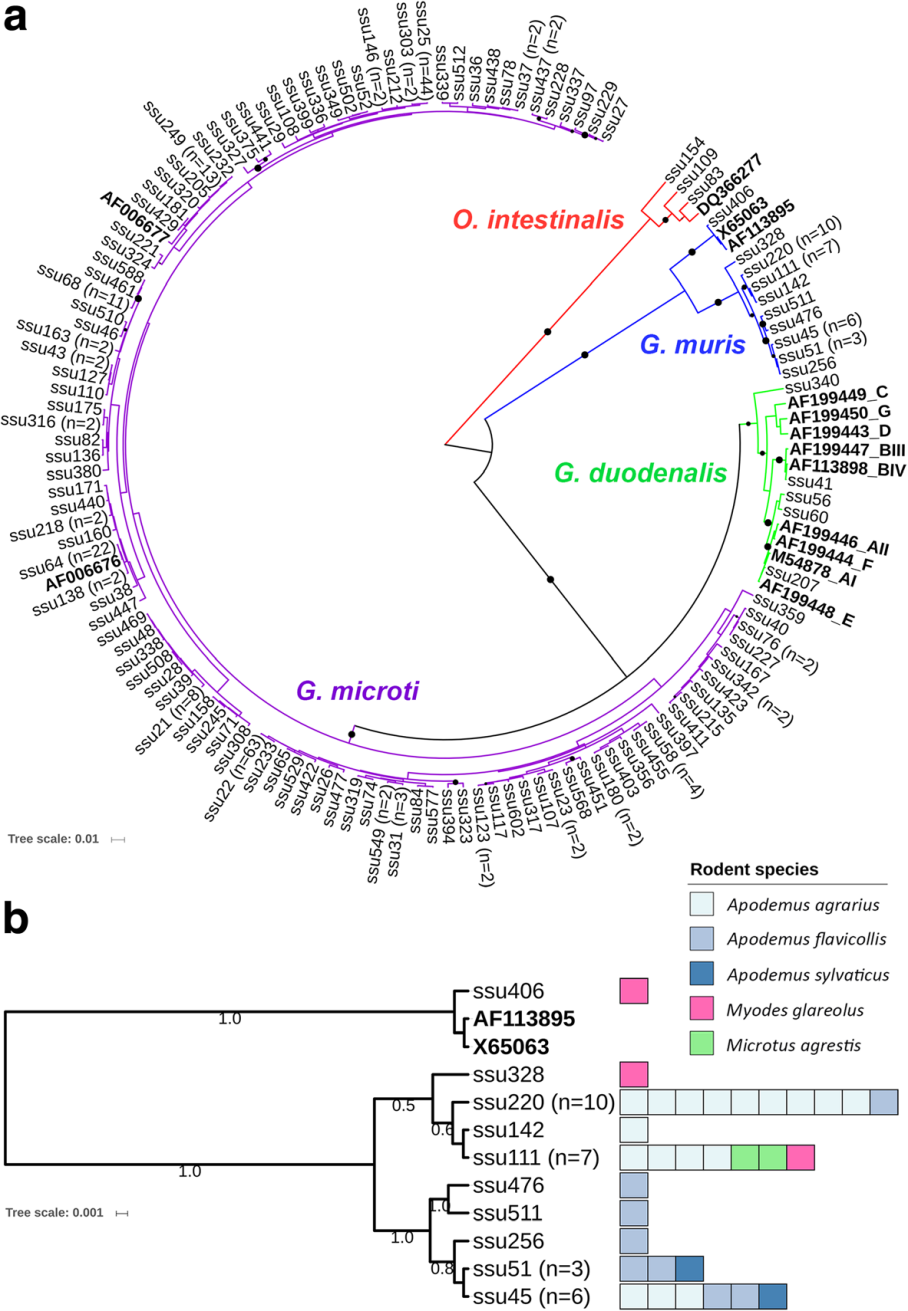
Bayesian phylogenetic analysis of unique *SSU* rDNA sequence fragments. Sequences of representative samples are shown and number of samples with identical sequences is given in brackets. Maximum likelihood analysis based on PhyML resulted in similar trees (not shown). **a** Unrooted phylogenetic tree comprised of 106 unique *SSU* rDNA sequences that have been classified as *G. microti* (purple clade), 10 unique sequences classified as *G. muris* (blue clade), 5 sequences classified as *G. duodenalis* (green clade) and 3 sequences classified as *O. intestinalis* (red clade), a sister lineage of *Giardia* spp. Reference sequences (GenBank accession numbers) of *O. intestinalis*, *G. muris*, *G. duodenalis* and *G. microti* are marked in bold. Posterior probabilities ≥ 0.5 are illustrated by black dots (proportionally increasing in size). **b** Unrooted phylogenetic tree of the 10 unique *G. muris* sequences and 2 references (GenBank: AF113895, X65063). Only posterior probabilities ≥ 0.5 are highlighted. Rodent species and number of samples from which unique sequences derived are illustrated in color bars (one square size represents one animal). Testing for significant phylogeny-trait correlations within the *G. muris* subgroup for host distribution clustering using the programme BaTS [51] revealed the following *P*-values: *A. agrarius* ≤ 0.001, *A. flavicollis* = 0.079

**Fig. 2:**
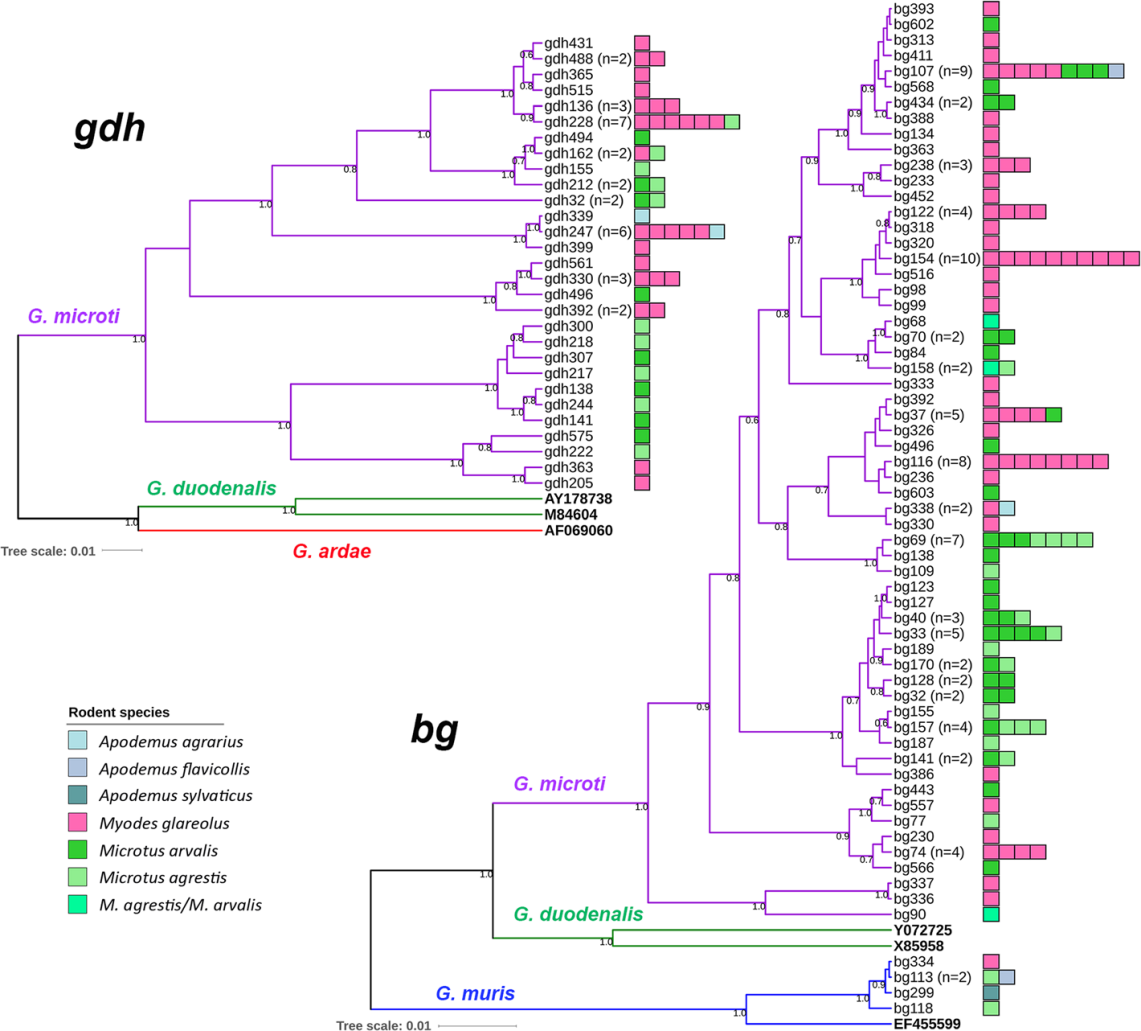
Bayesian phylogenetic analysis of unique *gdh* and *bg* sequence fragments. Sequences of representative samples are shown and number of samples with identical sequences is given in brackets. Unrooted phylogenetic trees comprised of 29 unique *gdh* and 59 unique *bg* sequences that have been classified as *G. microti* (purple clade) and 4 unique *bg* (and none *gdh*) sequences classified as *G. muris* (blue clade). Further reference sequences (GenBank accession numbers) for *bg* were included: *G. muris* (EF355599) and *G. duodenalis* (X85958, assemblage AI; Y072725, assemblage B; green clade). References for *gdh* included *G. duodenalis* (M84604, assemblage AI; AY178738, assemblage B; green clade) and *G. ardae gdh* (AF069060; red clade) sequences. Reference sequences are marked in bold. Only posterior probabilities ≥ 0.5 are highlighted. Rodent species and number of samples from which unique sequences derived are illustrated in color bars (one square size represents one animal). Maximum likelihood analysis based on PhyML resulted in similar trees (not shown). Testing for significant phylogeny-trait correlations within the *G. microti* subgroup for host distribution clustering using the programme BaTS [51] revealed the following *P*-values: *gdh*, *Myodes* = 0.079, *Microtus* ≤ 0.001; *bg*, *Myodes ≤* 0.001, *Microtus ≤* 0.001

Sequence analysis was performed first on 317 *SSU* rDNA sequences (277 *G. microti*, 5 *G. duodenalis*, 32 *G. muris* and 3 *O. intestinalis*) for which a complete sequence from both amplicon DNA strands was available (see Additional file 3: Figure S2 for dendrogram of Bayesian phylogenetic analysis of all *SSU* rDNA samples, including corresponding data of host species, locality, season, habitat and year of collection). Unique sequences were deduced from this data set and analyzed for possible phylogenetic relatedness which indicated high divergence within *G. muris* and *G. microti* sequences (Fig. 1). This prompted us to also type samples at the *gdh* and *bg* gene loci by established methods [5, 7] (Fig. 2).

Within the 32 *G. muris SSU* rDNA sequences, 10 unique sequence types were identified with four types represented by ≥ 3 identical sequences. Distance analysis revealed that 31 sequences differed by 0–10 single nucleotide polymorphisms (SNPs) to each other over a fragment length of 245–247 base pairs (bp). In contrast, one sample (ssu406, from *M. glareolus*) differed by 19–26 SNPs to these 31 sequences and this sequence was identical to the *G. muris* sequence AF113895 that was used for reference. The separation of the 31 sequences deduced here from current *G. muris* GenBank entries AF113895 and X65063 was also strongly supported by phylogenetic analyses that revealed a separation into two newly identified sequence clusters. Each of these sequence clusters was preferentially detected in different *Apodemus* hosts as determined by using phylogenetic trait analysis (Fig. 1b). Analysis of *bg* sequences identified five *G. muris* sequence types for this typing fragment. These sequences were also separated from *bg* GenBank sequence EF455599 used as reference further supporting the existence of phylogenetically distinguishable subgroups within the *G. muris* population (Fig. 2). No *gdh* sequences could be amplified of *G. muris* DNA containing samples and, notably, also no database entry exists for this gene.

*Giardia microti SSU* rDNA differed by 0–17 SNPs to each other over a fragment length of 250 bp. Within the *G. microti* samples, 106 unique sequences were identified, including 6 types represented by 8, 11, 13, 22, 44 and 63 identical sequences, respectively. The phylogenetic analysis is compatible with a genetic substructure of the population as previously suggested [13] although, expectedly, support of nodes was low due to the short sequence fragment used for the analysis (Fig. 1a). However, analysis of 29 *G. microti gdh* (unique sequence types deduced from a respective set of 49 total *G. microti* sequences) and 59 *G. microti bg* (deduced from a total set of 118 *G. microti* sequences of this locus) sequences strongly supports the existence of different phylogenetic subgroups in *G. microti* (Fig. 2). Testing for a significant association with host distribution (*Microtus vs Myodes*) of the phylogenetic subgroups using phylogenetic trait analysis indicated that subgroups are preferentially harbored by one of the two host genera (Fig. 2).

## Discussion

Rodents investigated in this study belonged to mice of the genus *Apodemus* (*A. agrarius*, *A. flavicollis* or *A. sylvaticus*) or to voles of the genus *Microtus* (*M. agrestis* or *M. arvalis*) or *Myodes* (*M. glareolus*). The overall occurrence of *Giardia* spp. in these animals was very high. *Apodemus* spp. were mainly infected with *G. muris*, whereas *G. microti* was predominantly found in *Microtus* spp. and *M. glareolus*. Our data argue that *G. muris* and *G. microti* subdivide into several phylogenetically distinguishable subgroups, each of which appears to be preferentially harbored by species of a particular rodent host genus. Only a low proportion of samples (1.4%) contained *G. duodenalis* assemblages A or B. This implies a very low potential risk for transmission of zoonotic *Giardia* types from wild rodents in Germany.

In contrast, several previous studies estimated a higher potential risk for zoonotic transmission from *Giardia*-infected wild rodents [17, 19, 24, 26, 31–34]. However, in only a few of these reports molecular analysis was performed to identify the underlying *Giardia* species. These studies concerned very different geographical regions and covered a variety of different rodent species: for example, *G. duodenalis* assemblages G and B in mice (*Mus* spp.) and rats (*Rattus* spp.) [17, 20], *G. duodenalis* assemblage B and *G. microti* in muskrats (*O. zibethicus*) [9, 13], and *G. microti* in prairie voles (*M. ochrogaster*) [13]. A study from Sweden found *G. muris* and *G. duodenalis* assemblage G in *M. musculus* and *R. norvegicus*, and *G. microti* in the one *A. flavicollis* analyzed, but no evidence for zoonotic *G. duodenalis* genotypes [15]. In contrast, beavers (*Castor* spp.) [9, 19] and pet chinchillas (*Chinchilla lanigera*) in particular [35], were found to harbor *G. duodenalis* assemblage B, indicating a potentially higher risk for zoonotic transmission associated with these host species. In summary, these and the present data highlight the need for local molecular typing analyses to estimate the actual zoonotic risk for *Giardia* infections emanating from a particular rodent host.

Previous studies from Poland also demonstrated high *Giardia* spp. prevalences in *A. flavicollis*, *M. glareolus* and *M. arvalis* [36, 37] and, in agreement with our results, showed lower parasite cyst loads in samples of *Apodemus vs* those from *Microtus* and *Myodes*. Systematic typing was not performed in these investigations, but four of the *Microtus* and *Myodes* samples were exemplarily typed and similar to our study revealed *G. microti* [38]. In this context it should be noted that fecal excretion of *Giardia* spp. is often not uniform. Therefore, quantitative data on individual time points should be interpreted carefully. However, it is assumed that such effects are averaged out when analyzing aggregated data as that presented here where the means of different groups were compared. Future studies will be necessary to investigate whether host-parasite sequence type relationships as reported here are a consistent finding also in other rodent populations.

Many studies of *Giardia* spp. in wild rodents used microscopy-based detection of parasite cysts and subsequently attempted to type *Giardia* by sequencing of PCR amplicons of one or a few marker genes. Often this approach was reported to be inefficient which suggests shortcomings in typing protocols. For example, a recent report on 12 rodent samples (ten *R. norvegicus*, two *M. musculus*) showed that *G. duodenalis* assemblage G could be typed at all four tested genomic loci (*bg*, *tpi*, *gdh*, *SSU* rDNA), but *G. muris* and *G. microti*-like samples only at the *SSU* rDNA locus [18]. Primer mismatches that may negatively affect PCR efficacy or low target DNA amounts that may reduce assay sensitivity could be possible reasons. In agreement with these data we also observed lower typing efficiency at *bg* and *gdh* loci in comparison to the *SSU* rDNA locus. We also recognized mismatches of published primers [4, 9, 13, 28] in the *G. muris SSU* rDNA reference sequences and, therefore, modified primer sets for our semi-nested *SSU* rDNA PCR assay. Modification resulted in higher positivity rates in particular for *G. muris* containing samples. In samples of *Microtus* spp. and *Myodes* spp., species predominantly infected with *G. microti*, the effect was much less pronounced. Thus to complement IFA data, we consider the semi-nested PCR at the *SSU* rDNA locus as the most reliable approach to detect the corresponding *Giardia* species. This approach will even detect non-*Giardia* species represented by *O. intestinalis*. Alternative PCR methods to discriminate *Giardia* spp. and *G. duodenalis* assemblages such as the recently developed tests that amplify *5.8S* rDNA or internal transcribed spacers (ITS) of ribosomal gene loci [38] may also be suitable but have not been evaluated in this study. PCR and sequencing of *bg* and *gdh* loci was more appropriate though for subtyping and description of population structure due to higher sequence heterogeneity. Because only two *bg* and no *gdh* sequences were deposited in public databases for *G. muris* and none of both for *G. microti* our work significantly enlarges the database which will greatly facilitate future comparative studies.

Our analysis not only confirmed that *G. microti* is more closely related to the *G. duodenalis* complex than to *G. muris* [13] but allowed also to distinguish genetic subgroups and provides evidence that these appear to preferentially infect different hosts. The existence of such subgroups was previously suggested for *G. microti* based on a very limited number of ribosomal RNA gene sequences [13] and is now supported by other markers through our analysis. This allows the interpretation that possibly various assemblages exist within *G. muris* and *G. microti* populations comparable to what has been described for *G. duodenalis*. Our study further indicates that *G. muris* also can be divided in sub-types that are preferentially associated with different hosts, in this case illustrated by *A. agrarius* and *A. flavicollis*. Most of the *G. muris SSU* rDNA sequences newly described were highly divergent from the two published sequences used as references. Both of the latter were derived from samples of non-European rodents [39, 40]. The concept of non-uniformity of *G. muris* is further supported by *bg* sequences, in spite of the low PCR efficiency at this locus. Further studies are warranted to determine the broader context and significance of phylogenetic relationships (e.g. host range, localities) within *G. muris* and *G microti*. This will require improved typing tools.

We also identified by semi-nested PCR at the *SSU* rDNA locus three sequences from *O. intestinalis*, a sister lineage from *Giardia* spp. known to infect wild rodents and together with *Giardia* spp. form the subfamily Giardiinae [30]. The life-cycle of *O. intestinalis* is not well characterized and, in particular, information about the morphology and antigenicity of cysts is scarce. All three samples (two *Microtus*, one *Myodes*) were also positive in the qPCR analysis and two samples in the IFA analysis. The *bg* sequence typing was also successful in the latter two samples and revealed *G. microti*. We therefore consider it likely that co-infections of *G. microti/O. intestinalis*, with a dominance of *O. intestinalis*, occurred in the respective hosts and that sequence similarity at the *SSU* rDNA locus of *Octomitus* spp. to *Giardia* spp. resulted in detection of the former. This is also corroborated by the high sequence similarity of the primer sequences used in the current study with published reference and primer sequences of the *SSU* rDNA sequence of *O. intestinalis* [30]. Co-infections may also occur with *G. microti* and *G. muris* which would impact our interpretations. However, we consider it unlikely that co-infections with different *Giardia* species are common in our sampled rodent population because, if true, mixed sequences should have been observed frequently.

## Conclusions

In the present study, *G. muris* was found to be present mainly in *Apodemus*, and *G. microti* mainly in *Microtus* and *Myodes* hosts. Furthermore, our findings argue for a very low prevalence of zoonotic *G. duodenalis* assemblages in wild rodents in Germany. Evidence is provided that *G. muris* and *G. microti* subdivide into phylogenetically distinct subgroups that each prefers species of a particular rodent host genus (in the case of *G. microti*) or family (in the case of *G. muris*). The study also expands the database of sequences relevant for sequence typing of *G. muris* and *G. microti* isolates. In future studies this will greatly help to analyze the population structure of these parasites in more detail.

## Methods

### Sample collection

Samples were collected during spring, summer and autumn in 2011 and 2012. Samples collected during field work in four Federal states in Germany were utilized *via* the German Network “Rodent-borne pathogens” [41]. Rodents were live trapped in “grassland (GL)” and “forest (F)” sites named after the nearby town of the study sites (German Federal State in brackets): Billerbeck (North Rhine-Westphalia, NRW), Weissach (Baden-Wuerttemberg, BW), Gotha (Thuringia, TH), Jeeser (Mecklenburg-Western Pomerania, MV). A further study site close to Jeeser (GL) was Fuhlendorf-Darß, Saal (MV). In addition, rodents were sampled at six study sites in TH: Kammerforst (Treben) (F), Krahnberg (Gotha) (GL), Leinawald (Altenburg) (F), Pahnaer Holz (Eschefeld) (F), Phönix Ost (Schnauderhainichen) (F), Schaderode (Erfurt) (GL). The latter sites were in reforestation areas and classified according to their “best-match” to GL or F.

Fecal samples (1–2 pellets per animal) were collected from 577 individual animals and preserved in 1 ml H_2_O supplemented with a cocktail of antibiotics in order to inhibit bacterial growth. Samples were shipped at room temperature and stored at 4 °C until further processing (storage time between 1 and 10 months). Rodent species were identified mainly by field inspection, in some cases molecular typing was performed on fecal samples using cytochrome *b* as a target gene as described earlier [42].

### IFA test

All samples were examined using the MERIFLUOR® *Cryptosporidium*/*Giardia* test (Meridian Bioscience, Luckenwalde, Germany) following the manufacturer’s protocol and results were qualitatively assigned as being positive or negative for *Giardia*. In addition, samples were used for a semi-quantitative assignment of cyst numbers in the microscopic sample, which approximates 10 μl of volume: +, 1–10 cysts; ++, 11–50 cysts; +++, > 50 cysts.

### DNA isolation

Genomic DNA was extracted from samples using one of the two commercial kits following the protocols of the manufacturers: QIAamp DNA Stool Mini Kit (Qiagen, Hilden, Germany) or Maxwell® 16 FFPE Plus LEV DNA Purification Kit (Promega, Mannheim, Germany). The final elution volume was 70 μl. Both kits have been tested and performed similarly in subsequent PCR applications. The amount of nucleic acid in samples was photometrically estimated at OD_260_.

### qPCR

All samples were tested using a TaqMan-based *Giardia* specific qPCR as earlier described [27, 43] with minor modifications. Briefly, a 62 bp fragment of *SSU* rDNA was amplified in a total PCR volume of 25 μl [12.5 μl Maxima Probe/ROX qPCR Master Mix 2× (ThermoFisher Scientific, Schwerte, Germany); 3 μM of each primer (*Giardia*-80F and *Giardia*-127R), 1.5 μM of double labeled probe (*Giardia*-105T, 5'FAM, 3'BHQ1) and 1 μl of the DNA]. Amplification cycles consisted of 95 °C for 15 min followed by 40 cycles at 95 °C for 15 s, 60 °C for 30 s, and 72 °C for 30 s. Amplification, detection, and data analysis were performed with a Stratagene Mx3000P cycler and according MxPro software (both from Agilent Technologies, Waldbronn, Germany). Titration experiments with DNA from *G. duodenalis* trophozoites (WB6, ATCC 50803) revealed a detection limit (analytical sensitivity) in our technical setting of approximately one trophozoite (equals approximately four genome equivalents) in the PCR. Analytical specificity was calculated as 98% (calculated from 49 control PCRs of which one was slightly positive with a Ct-value of 38.5). Inhibition was not found to be an issue in the PCR analyses (Only one of 35 tested samples showed a slight inhibition with a delta-Ct value > 2 in a test set with internal amplification control).

We also used the Ct-value of the qPCR analysis as a proxy for parasite numbers. We assumed that target DNA integrity is similar in the samples’ fecal matrix of all three rodent genera and that PCR efficacies were equal, although we were not able to formally test these assay parameters.

### Typing PCR and sequencing

A fragment of the *SSU* rRNA gene was amplified using a combination of previously described and modified PCR protocols [4, 28]. A nested PCR protocol was used with the initial primer pair RH11-derivates (equal mix of 5'-CAT CCG GTC GAT CCT GCC-3' and 5'-CAT CCG GTT GAT CCT GCC-3') and Gia2150c (5'-CTG CTG CCG TCC TTG GAT GT-3') amplifying a 497 bp product and a secondary primer pair for semi-nested PCR RH11-derivates (equal mix of 5'-CAT CCG GTC GAT CCT GCC-3' and 5'-CAT CCG GTT GAT CCT GCC-3') and RH4-derivates (equal mix of 5'-AGT CGA ACC CTG ATT CTC CGC CAG G-3' and 5'-AGT CAA ACC CTG ATC CTC CGC CAG G-3' and 5'-AGT CGA ACC CTG ATT CTC CGT CAG G-3') amplifying a 293 bp fragment. The PCR consisted of 1 μl of DNA for the primary reaction and of 2 μl of primary PCR mix for the nested PCR, 200 μM dNTPs, 1× PCR Mango*Taq* buffer containing 3 mM MgCl_2_ (Bioline, Luckenwalde, Germany), 2.5 U of Mango*Taq* polymerase (Bioline), and 200 nM of each primer (-mix) in 25 μl in the primary PCR and 50 μl reaction in the nested PCR. The reactions were performed for 35 cycles under following conditions: 1st PCR (94 °C for 45 s, 55 °C for 30 s and 72 °C for 45 s) and nested-PCR (94 °C for 45 s, 59 °C for 30 s and 72 °C for 45 s). They were run with an initial hot start (95 °C for 5 min) and a final extension at 72 °C for 7 min.

Fragments of the *bg* and *gdh* genes were amplified according to previously described nested-PCR protocols [5, 7]. Briefly, PCRs were run in a total volume of 50 μl and included 1–2 μl target DNA, 200 μM dNTPs, 1× PCR Mango*Taq* buffer containing 3 mM MgCl_2_ (Bioline), 2.5 U of Mango*Taq* polymerase (Bioline), and 200 nM of each primer. The reactions were performed for 35 cycles using following conditions: 1st PCR (94 °C for 45 s, 65 °C (for *bg*) and 56 °C (for *gdh*) for 30 s and 72 °C for 45 s) and nested-PCR (94 °C for 45 s, 65 °C (for *bg*) and 56 °C (for *gdh*) for 30 s and 72 °C for 45 s). Final extension was done for 7 min at 72 °C.

All PCR products were analyzed by electrophoresis on 1.2% agarose gels and visualized after GelGreen (Biotium, Fremont, USA) staining. PCR products of the expected size were purified by using the ExoSAP-IT® For PCR Product Cleanup kit (ThermoFisher Scientific). Sequencing reactions were performed in both directions using BigDye 3.1 sequencing reagents (Applied Biosystems) and primer specific annealing temperatures as stated above. The *SSU* rDNA sequences were compared to selected reference sequences in order to identify *Giardia* spp. and *G. duodenalis* assemblages by using built-in applications of Geneious software 9.1. (Biomatters Ltd., Auckland, New Zealand). The following reference sequences were used: *G. muris* (X65063, AF113895), *G. microti* (AF006676, AF006676), *G. duodenalis* assemblage A (M54878), *G. duodenalis* assemblage B (AF199447), *G. duodenalis* assemblage C (AF199449), *G. duodenalis* assemblage D (AF199443), *G. duodenalis* assemblage E (AF199448), *G. duodenalis* assemblage F (AF199444), *G. duodenalis* assemblage G (AF199450), *O. intestinalis* (DQ366277). For sequence analysis of *bg* and *gdh* the following reference sequences were used: *G duodenalis* assemblage B (AY072725, AY178738), *G. duodenalis* assemblage A (X85958, M84604), *G. muris bg* (EF455599), *G. ardae gdh* (AF069060).

Some of the obtained sequences were analyzed to identify the most similar sequence deposited in the GenBank public database using the built-in Basic Local Alignment Search Tool (BLAST) algorithms (http://blast.ncbi.nlm.nih.gov/Blast.cgi). Sequence names are deduced from a unique running sample number preceded by an abbreviation of the sequenced genes (e.g. ssu349 = *SSU* rDNA sequence of rodent isolate number 349). Nucleotide sequences generated in this study have been deposited in the GenBank database under accession numbers KY114167-KY114486 (*SSU* rDNA) and MG676938-MG677109 (*bg* and *gdh*). Accession numbers of each sequence are listed in Additional file 4: Table S2.

### Phylogenetic analysis

Alignments of completely sequenced PCR products (without primer sequences) and respective regions of the reference sequences were produced using MUSCLE [44] integrated subroutine of Geneious version 9.1. (Biomatters Ltd.). Sequences containing polymorphic positions, a well-known phenomenon in *G. duodenalis* [45], were included in the analysis when the sequencing data of both strands was available. Bayesian analysis of sequence alignments was performed by using the BEAUti and BEAST software packages [46]. PhyML analysis was done with ATGC online tool PhyML 3.0 [47] using the Smart Model Selection tool to select the best substitution model and subsequent PhyML analysis using the best substitution model with Subtree-Pruning-Regrafting (SPR) tree searching and bootstrap performance of 100 [48]. Trees were annotated using the iTOL online tool [49, 50]. Phylogenetic trait analysis was done using the program BaTS [51].

### Statistical analysis

Data were organized in a spread sheet and subsequently imported into the statistics software package STATA 14.1 (StataCorp, College Station, USA). Proportions were calculated and analyzed for binomial exact 95% confidence intervals (95% CI). To test for any difference between proportions of groups the Fisher-Freeman-Halton test was used [52]. For comparison between groups, the two-sided Fisher’s exact test was used and a multiple correction using the Bonferroni-Holm procedure was applied and adjusted *P*-values are reported [53]. A *P*-value of ≤ 0.05 was considered statistically significant. Due to the low number of cases we have chosen not to use multivariate analyses using logistic regression models. For qPCR data (Ct values) the median and min/max values of groups were calculated and non-parametric Kruskal-Wallis test was performed followed by Dunn’s test of multiple comparisons to assess statistical significance using the software package GraphPad Prism 7.03 (a *P*-value of ≤ 0.05 was considered statistically significant).

## Additional files

Additional file 1: **Figure S1.** Map of Germany with study sites where wild rodents were captured and sampled. Rodents were captured at 11 study sites that were subdivided into five regions (refer to the color coding) from four German federal states. Please refer to Table 1 for further details on captured animals from each site. (TIFF 780 kb)

Additional file 2: **Table S1.** Statistical comparison (*P*-values) between groups as depicted in Table 1 using Fisher’s exact test followed by multiple testing correction (Bonferroni-Holm procedure). (DOCX 17 kb)

Additional file 3: **Figure S2.** Bayesian phylogenetic analysis of all *SSU* rDNA sequence fragments. Unrooted phylogenetic tree comprised of 317 *SSU* rDNA sequences (277 *G. microti*, purple clade; 5 *G. duodenalis*, green clade; 32 *G. muris*, blue clade and 3 *O. intestinalis*, red clade). Reference sequences (GenBank accession numbers) of *O. intestinalis*, *G. muris*, *G. duodenalis* and *G. microti* are included (uncoloured sequence names). Posterior probabilities ≥ 0.5 are illustrated by black dots (proportionally increasing in size). Sequence names are color coded (colored ranges) according to the rodent host. Further color coding (inner to outer layer) was introduced according to locality [1 to 11: 1 (“Billerbeck”), 2 (“Gotha”), 3 (“Krahnberg”), 4 (“Schaderode”), 5 (“Jeeser”), 6 (“Saal”), 7 (“Kammerforst”), 8 (“Leinawald”), 9 (“Pahnaer Holz”), 10 (“Phönix Ost”), 11 (“Weissach”); see also Additional file 1: Figure S1 and Table 1], season (spring, summer, autumn), habitat (F = “forest”, GL= “grassland”) and year of sample collection (2011, 2012). Maximum likelihood analysis based on PhyML resulted in a similar tree (not shown). (TIFF 2598 kb)

Additional file 4: **Table S2.** Accession numbers of nucleic acid sequences generated in the present study. (XLSX 26 kb)

## Abbreviations

*tpi*: triosephosphate isomerase
*gdh*: glutamate dehydrogenase
*bg*: beta-giardin
*SSU* rDNA: small-subunit ribosomal RNA gene
IFA: immunofluorescence assay
qPCR: real time-PCR
GL: grassland
F: forest
NRW: North Rhine-Westphalia
BW: Baden-Wuerttemberg
TH: Thuringia
MV: Mecklenburg-Western Pomerania
SNPs: single nucleotide polymorphisms
