# Supplementary figures and images for: Occurrence and distribution of Giardia species in wild rodents in Germany
Source: Parasit Vectors. 2018 Mar 27;11:213. doi: 10.1186/s13071-018-2802-z (PMC5870188; doi:10.1186/s13071-018-2802-z)

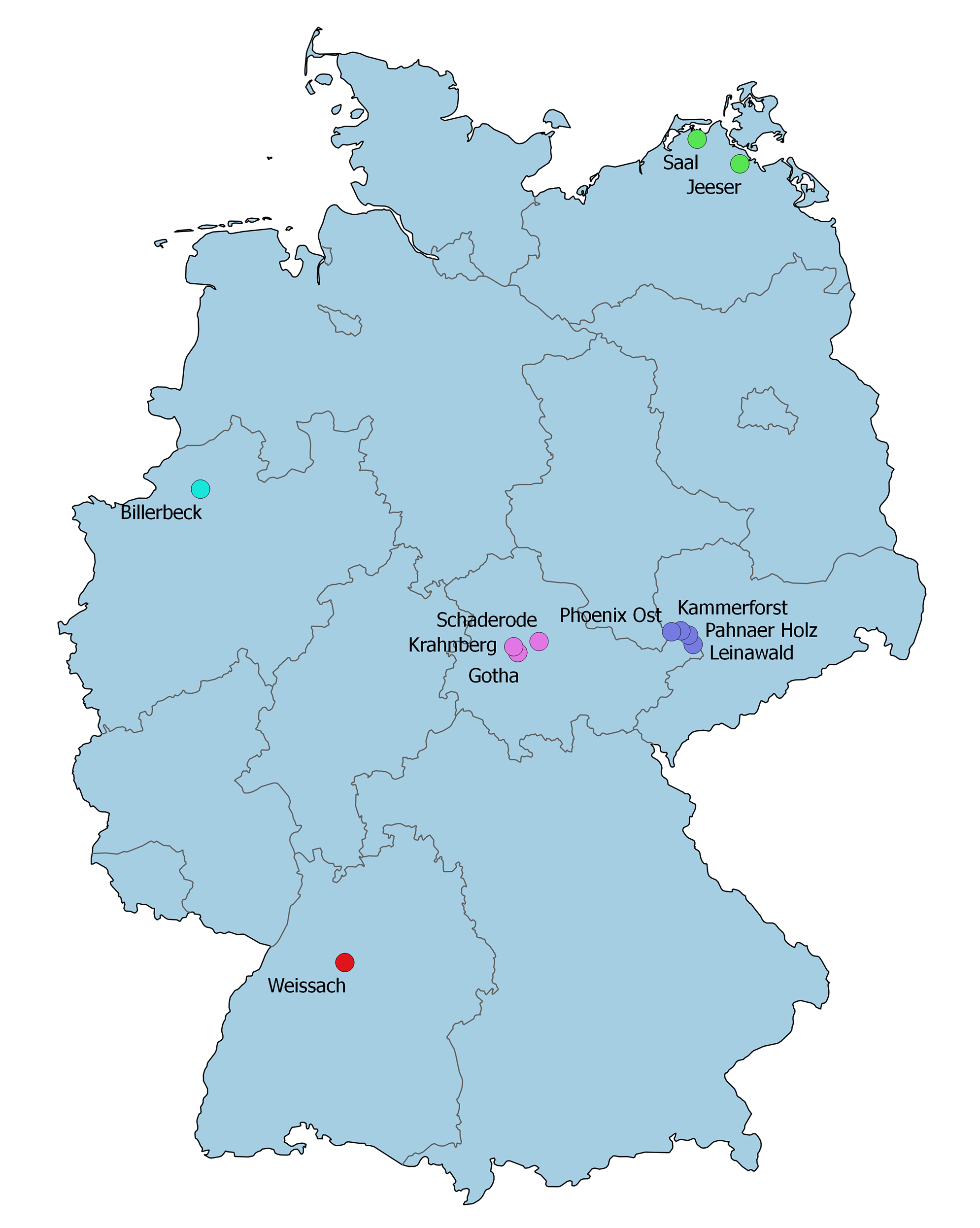

Supplement: Supplementary file 1 — Figure S1. Map of Germany with study sites where wild rodents were captured and sampled. Rodents were captured at 11 study sites that were subdivided into five regions (refer to the color coding) from four German federal states. Please refer to Table 1 for further details on captured animals from each site. (TIFF 780 kb) [file 13071_2018_2802_MOESM1_ESM.tif]

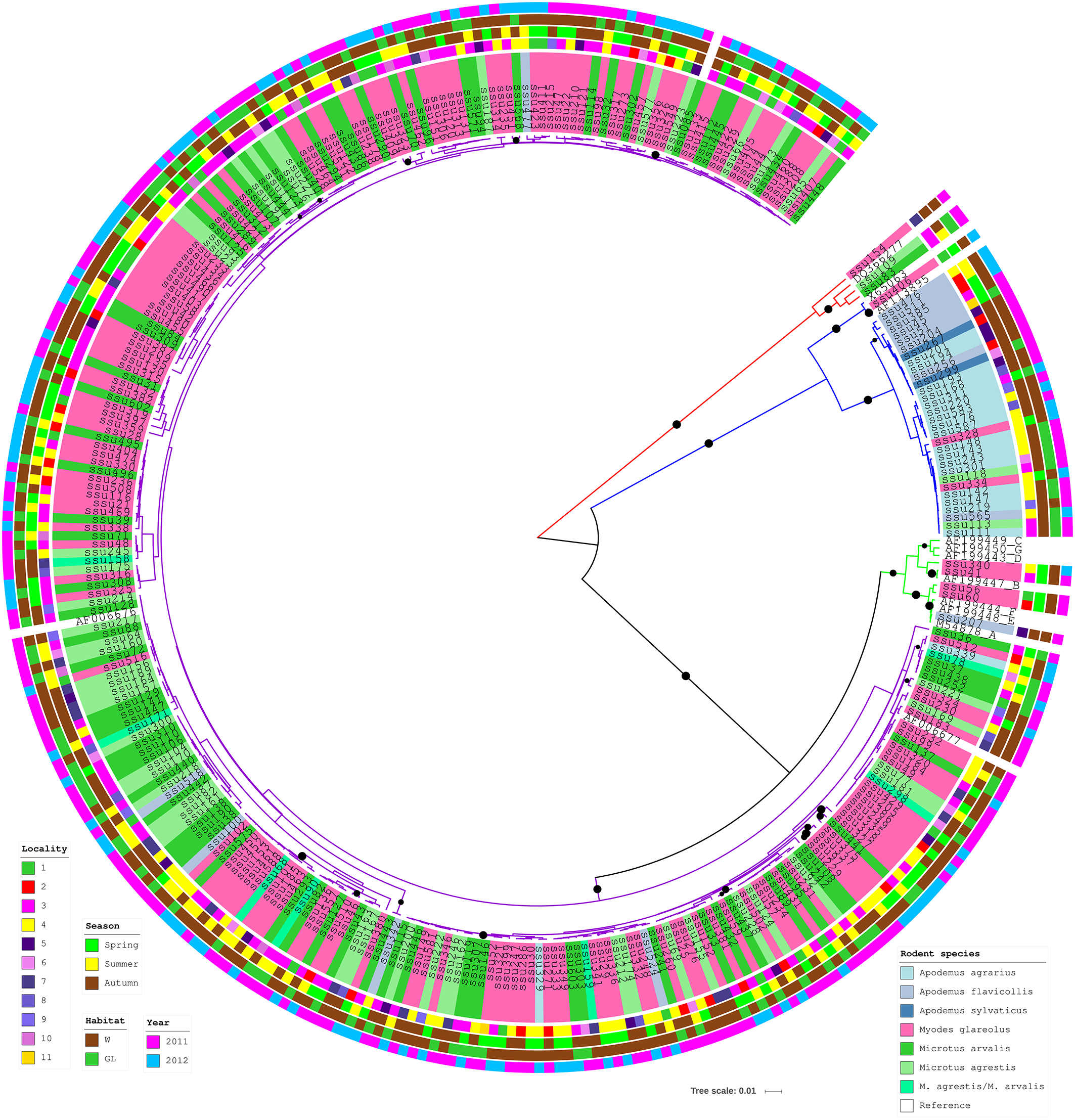

Supplement: Supplementary file 3 — Figure S2. Bayesian phylogenetic analysis of all SSU rDNA sequence fragments. Unrooted phylogenetic tree comprised of 317 SSU rDNA sequences (277 G. microti, purple clade; 5 G. duodenalis, green clade; 32 G. muris, blue clade and 3 O. intestinalis, red clade). Reference sequences (GenBank accession numbers) of O. intestinalis, G. muris, G. duodenalis and G. microti are included (uncoloured sequence names). Posterior probabilities ≥ 0.5 are illustrated by black dots (proportionally increasing in size). Sequence names are color coded (colored ranges) according to the rodent host. Further color coding (inner to outer layer) was introduced according to locality [1 to 11: 1 (“Billerbeck”), 2 (“Gotha”), 3 (“Krahnberg”), 4 (“Schaderode”), 5 (“Jeeser”), 6 (“Saal”), 7 (“Kammerforst”), 8 (“Leinawald”), 9 (“Pahnaer Holz”), 10 (“Phönix Ost”), 11 (“Weissach”); see also Additional file 1: Figure S1 and Table 1], season (spring, summer, autumn), habitat (F = “forest”, GL= “grassland”) and year of sample collection (2011, 2012). Maximum likelihood analysis based on PhyML resulted in a similar tree (not shown). (TIFF 2598 kb) [file 13071_2018_2802_MOESM3_ESM.tif]
