# Supplementary material for: Occurrence and distribution of Giardia species in wild rodents in Germany
Source: Parasit Vectors. 2018 Mar 27;11:213. doi: 10.1186/s13071-018-2802-z (PMC5870188; doi:10.1186/s13071-018-2802-z)
Supplement: Supplementary file 2 — Table S1. Statistical comparison (P-values) between groups as depicted in Table 1 using Fisher’s exact test followed by multiple testing correction (Bonferroni-Holm procedure). (DOCX 17 kb) [file 13071_2018_2802_MOESM2_ESM.docx]

Additional file 2: Table S1. Statistical comparison (*P*-values) between groups as depicted in Table 1 using Fisher’s exact test followed by multiple testing correction (Bonferroni-Holm procedure).

|  | Analysis | Prevalence (%) | 95% CI | *M. glareolus* | *M. arvalis* | *M agrestis* | *A. sylvaticus* | *A. flavicollis* |
| --- | --- | --- | --- | --- | --- | --- | --- | --- |
| *A agrarius* | IFA | 22.9 | 10.4–40.1 | *P* ≤ 0.0001 | *P* ≤ 0.0001 | *P* ≤ 0.0001 | *P* = 0.586 | *P* = 1 |
|  | qPCR | 51.4 | 34.0–68.6 | *P* = 0.0005 | *P* = 0.015 | *P* = 0.0003 | *P* = 0.036 | *P* = 0.348 |
|  | *SSU* PCR | 68.6 | 50.7–83.1 | *P* = 1 | *P* = 1 | *P* = 0.302 | *P* = 0.165 | *P* = 0.049 |
| *A. flavicollis* | IFA | 23.7 | 11.4–40.2 | *P* ≤ 0.0001 | *P* ≤ 0.0001 | *P* ≤ 0.0001 | *P* = 0.586 |  |
|  | qPCR | 31.6 | 17.5–48.6 | *P* ≤ 0.0001 | *P* ≤ 0.0001 | *P* ≤ 0.0001 | *P* = 0.348 |  |
|  | *SSU* PCR | 34.2 | 19.6–51.3 | *P* = 0.009 | *P* = 0.075 | *P* = 0.002 | *P* = 1 |  |
| *A. sylvaticus* | IFA | 55.6 | 21.2–86.3 | *P* = 0.586 | *P* = 0.178 | *P* = 0.233 |  |  |
|  | qPCR | 0 | 0–33.6 | *P* ≤ 0.0001 | *P* = 0.0002 | *P* ≤ 0.0001 |  |  |
|  | *SSU* PCR | 22.2 | 2.8–60.0 | *P* = 0.177 | *P* = 0.023 | *P* = 0.002 |  |  |
| *M. agrestis* | IFA | 87.7 | 77.2–94.5 | *P* = 0.586 | *P* = 1 |  |  |  |
|  | qPCR | 98.3 | 90.9–100 | *P* = 0.316 | *P* = 0.348 |  |  |  |
|  | *SSU* PCR | 86.7 | 75.4–94.1 | *P* = 0.004 | *P* = 0.442 |  |  |  |
| *M. arvalis* | IFA | 88.9 | 81.4–94.1 | *P* = 0.178 |  |  |  |  |
|  | qPCR | 90.7 | 83.5–95.4 | *P* = 1 |  |  |  |  |
|  | *SSU* PCR | 75.7 | 66.5–82.5 | *P* = 0.165 |  |  |  |  |
